# Down-Regulation of Honey Bee *IRS* Gene Biases Behavior toward Food Rich in Protein

**DOI:** 10.1371/journal.pgen.1000896

**Published:** 2010-04-01

**Authors:** Ying Wang, Navdeep S. Mutti, Kate E. Ihle, Adam Siegel, Adam G. Dolezal, Osman Kaftanoglu, Gro V. Amdam

**Affiliations:** 1 School of Life Sciences, Arizona State University, Tempe, Arizona, United States of America; 2 Department of Chemistry, Biotechnology, and Food Science, Norwegian University of Life Sciences, Aas, Norway; University of Illinois at Urbana-Champaign, United States of America

## Abstract

Food choice and eating behavior affect health and longevity. Large-scale research efforts aim to understand the molecular and social/behavioral mechanisms of energy homeostasis, body weight, and food intake. Honey bees (*Apis mellifera*) could provide a model for these studies since individuals vary in food-related behavior and social factors can be controlled. Here, we examine a potential role of peripheral *insulin receptor substrate* (*IRS*) expression in honey bee foraging behavior. *IRS* is central to cellular nutrient sensing through transduction of insulin/insulin-like signals (IIS). By reducing peripheral *IRS* gene expression and IRS protein amount with the use of RNA interference (RNAi), we demonstrate that *IRS* influences foraging choice in two standard strains selected for different food-hoarding behavior. Compared with controls, *IRS* knockdowns bias their foraging effort toward protein (pollen) rather than toward carbohydrate (nectar) sources. Through control experiments, we establish that *IRS* does not influence the bees' sucrose sensory response, a modality that is generally associated with food-related behavior and specifically correlated with the foraging preference of honey bees. These results reveal a new affector pathway of honey bee social foraging, and suggest that *IRS* expressed in peripheral tissue can modulate an insect's foraging choice between protein and carbohydrate sources.

## Author Summary

Food choice, food handling, and eating are aspects of food-related behavior that can become pathological, as seen in the public health problems of obesity and diabetes. Thus, the insight that molecules from the body can bind to brain cells and signal and change food-related behavior is of biomedical interest. One such molecule is insulin, which binds to cells via receptors attached to the insulin receptor substrate protein, IRS. Insulin-like systems are found in all multi-cellular animals, and receptors with IRS are found in many cell types, including fat and muscle where receptor-binding regulates glucose uptake. However, it is unknown whether signaling to these “peripheral” tissues, in contrast to brain, have behavioral consequences. We suppress the gene encoding for IRS protein in the fat tissue of honey bees, insects with advanced food-related behavior. In response, animals collected less carbohydrate-rich food (sugar-containing nectar) and biased their foraging toward pollen, a protein source. Sensory sensitivity to sugar influences honey bee foraging behavior, but we show that this sensitivity remained unaltered when the *IRS* gene was suppressed. This study identifies a new molecular pathway that may regulate food-hoarding in bee colonies and shows that food-related behavior can be influenced by insulin-like signaling to peripheral cells.

## Introduction

Multicellular animals have distinct energy demands but can modulate their growth and energy consumption in response to nutrient availability [1],[2]. This state of metabolic homeostasis is central to health and lifespan. Metabolic homeostasis is maintained by physiological feedback mechanisms that include the behavioral system [3]. The association between metabolic biology and behavior is of much interest since human food-choice and eating behavior contribute to many public health-issues such as obesity and diabetes [4]. In mammals, food-related behavior is influenced by several factors, including age [5], sex and reproductive physiology [6], genotype [7], sensory perception [8],[9], and environment or social setting [10]. Many of these factors interact in complex ways to affect behavior [11]–[13], and the underlying cause-effect relationships are challenging to test. However, similar relationships are found in highly manipulable insect models where metabolic biology shows considerable homology to mammalian systems [14].

Insect food-related behavior, as exemplified by individual foraging choice between a carbohydrate source (nectar) and a protein source (pollen), is studied in detail in honey bees (*Apis mellifera*) [15]–[17]. Honey bees are social insects organized in colonies with one reproductive queen and several thousands of largely sterile female helpers called workers [18]. Workers progress through an age-associated series of tasks that culminate in foraging activity when bees are 2–3 weeks old. As foragers, workers collect nectar, pollen, water and propolis, which are essential resources for colony growth and survival. Nectar and pollen are stored (hoarded) inside the nest and consumed as a function of colony needs. A worker can collect both nectar and pollen during a foraging trip, but she will often bias her collection toward one of these resources [19]. Bidirectional colony-level artificial selection for the amount of stored pollen (pollen-hoarding) resulted in high and low pollen-hoarding honey bees that are maintained as standard strains [17]. These strains are characterized by significantly different foraging behavior in workers: Similar to the wild type (unselected commercial stocks), high and low pollen-hoarding strain bees collect nectar, pollen or both during foraging trips, but high strain workers are more likely to collect pollen [16],[17],[20],[21].

Physiological, sensory and behavioral systems are tightly linked in animals [7],[8],[22], including insects [23]. As a likely consequence, bidirectional selection for pollen-hoarding affected not only foraging behavior, but also behavior-associated physiology such as circulating levels of vitellogenin (yolk protein precursor/behavioral affector molecule [15], [24]–[26]) and sensory systems (sucrose responsiveness [27]–[29]). Studies in wild-type honey bees have confirmed correlations as well as direct relationships between these traits [24],[29],[30]. Moreover, genome mapping has identified highly epistatic quantitative trait loci (QTL, *pln1*- *pln4*) that explain variation in honey bee foraging behavior and sucrose responsiveness [31]–[33]. The 95% confidence interval of the least gene-dense QTL, *pln4*, contains four genes. One is the *insulin receptor substrate* (*IRS*), which is an appealing positional candidate gene for regulation of honey bee behavioral physiology due to known interactions between the IIS pathway and food-related behavior [34],[35].

*IRS* genes encode for a conserved membrane-associated adaptor protein that is central to transduction of insulin/insulin-like signals (IIS) (reviewed by [36]). IIS pathways, including IRS proteins, are active in the central (neural) and peripheral (non-neural) tissues of eukaryotes and regulate metabolic responses to food-intake [2],[22],[37]. Central nervous system IIS (central IIS) can also coordinate eating behavior directly (reviewed by [38],[39]); e.g., following administration or natural secretion of insulin, elevated central IIS will change food-intake behavior [40]. In mammals, the increase in blood nutrient-levels after eating leads to enhanced synthesis and release of insulin from pancreatic β-cells, while insects release insulin-like peptides (ILPs) from neural cells [41]. The activity of pancreatic cells is further influenced by gastrointestinal hormones (incretins) and signals from the autonomic nervous system (reviewed by [42],[43]), whereas recent work in the fruit fly *Drosophila melanogaster* shows that humoral signals from peripheral fat body (insect functional homolog of mammalian liver and adipose tissue) can regulate ILP secretion in brain [44]. The *Drosophila* IRS homologue CHICO is crucial for IIS function in fly tissues including neural cells, and fly behavior is affected if central IIS is experimentally impaired [45]. Contrasting these and other findings about roles of central IIS in behavior, less is known on how behavior is influenced by peripheral IIS, i.e., signaling that is endogenous to peripheral tissues.

Here, we use honey bees to test the prediction that perturbation of peripheral IIS can affect food-related behavior. Experimental workers were obtained from the standard strains of high and low pollen-hoarding bees, while wild type was used to test the general validity of methods and select results. Pollen-hoarding strain bees were preferred as experimental animals because the set of well-defined phenotypic differences between them allow treatment effects and their interactions with genotype to become more readily apparent ([15],[24] and Discussion). Perturbation of peripheral IIS was achieved by RNA interference (RNAi)-mediated gene knockdown of *IRS* in fat body.

The results presented here show that food-related behavior can be influenced by changes in peripheral IIS: *IRS* RNAi, which reduced *IRS* expression levels in worker fat body but not in brain, biased bees to forage for the protein source, pollen. Our detailed analyses of genotype-specific behavioral patterns and established factors connected to variation in honey bee foraging behavior (*vitellogenin* gene expression, sucrose sensory sensitivity) point to distinct roles of *IRS* in regulation of worker foraging choice.

## Results

### Validation of peripheral *IRS* knockdown

Newly emerged (0–24 h old) adult workers from high and low pollen-hoarding strains were injected intra-abdominally [46],[47] with double-stranded RNA (dsRNA) against the only IRS-encoding gene in honey bees (GenBank XM_391985). This approach to RNAi targets honey bee fat body [30],[46],[48],[49] while being ineffective in brain [47],[50]. Knockdown was assessed relative to an established honey bee control procedure for non-specific effects of treatment or handling in RNAi experiments. This protocol requires injection of dsRNA toward a gene not found in the bee (a green fluorescent protein (GFP) encoding gene in vector, GenBank AF097553) [30],[48],[49]. The design was replicated twice by introducing workers into two separate host colonies.

#### Real-time quantitative reverse transcription PCR (qRT–PCR)

Transcript abundance was measured in fat body and brain when knockdown and control workers were 7 days old (n = 18).

**Figure 1:**
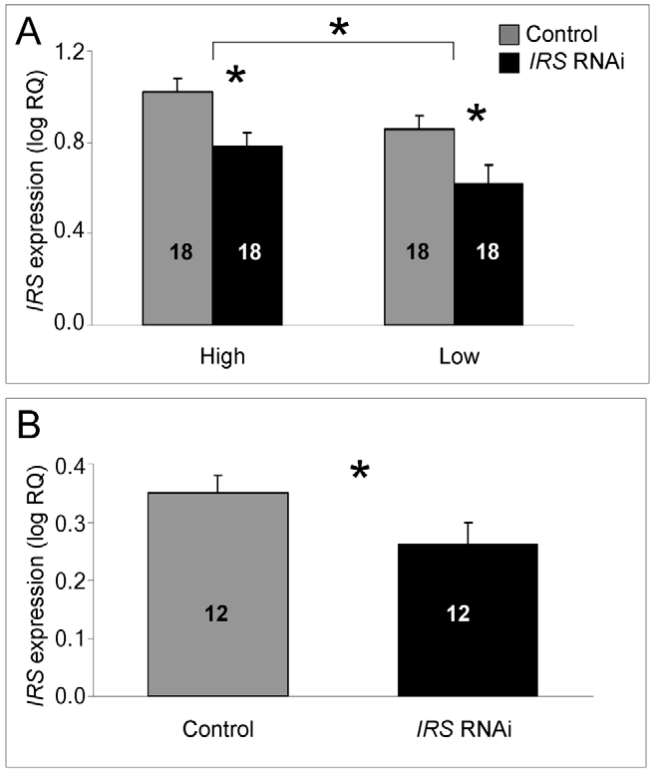
qRT–PCR validation of *IRS* RNAi in peripheral fat body. *IRS* mRNA levels in honey bee fat body, shown as log relative quantities (RQ). Controls were injected with double-stranded RNA (dsRNA) toward a green florescent protein (GFP) encoding gene, while *IRS* RNA interference (RNAi) was triggered by injection of dsRNA toward the only *IRS* encoding gene in honey bees. (A) Validation of *IRS* knockdown in high and low pollen-hoarding strain bees. Genotype had an independent and significant effect on *IRS* expression [asterisk and brackets on top of (A)]. (B) Validation of *IRS* knockdown in wild type. Asterisks indicate significance, p<0.05. Bars are means ± s.e. Sample sizes are given inside bars. (A) versus (B) were quantified with different calibrator samples; Y-axes cannot be directly compared.

In fat body, we could confirm that injection of *IRS* dsRNA triggered RNAi: *IRS* transcript levels were significantly reduced when summing over the entire data from the two genotypes and host colonies (factorial ANOVA: treatment, F_(1,63)_ = 11.4808, p = 0.0012). The dataset also revealed that *IRS* expression was influenced by genotype *per se*, but not by host colony environment (factorial ANOVA: genotype, F_(1,63)_ = 4.9416, p = 0.0298; colony, F_(1,63)_ = 0.5440, p = 0.4635). High pollen-hoarding strain workers had significantly higher *IRS* mRNA levels than low strain bees (Figure 1A). The RNAi effect, furthermore, was independently significant within both strain genotypes (Fisher's leased significant difference test (LSD): high strain, p = 0.0108; low strain, p = 0.0359), while there was no interaction-effect between treatment and genotype (F_(2,63)_ = 0.0037, p = 0.9514). Validation of the protocol in wild-type worker fat body (n = 12, Figure 1B) established that our approach to *IRS* knockdown was robust and not restricted to the selected strains (one-tailed Student's t-test, T _(1,21)_ = 1.8951, p = 0.0356).

**Figure 2:**
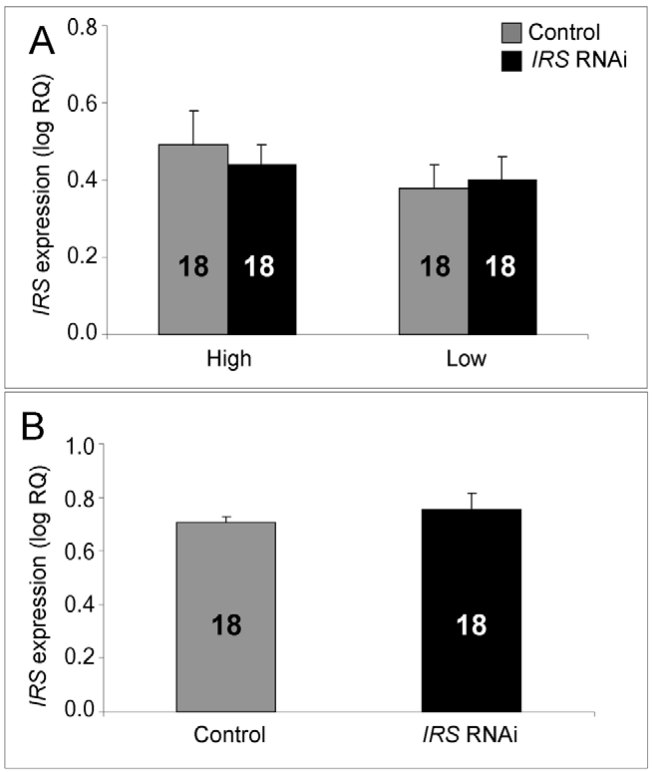
qRT–PCR confirms absence of *IRS* RNAi in brain. IRS dsRNA injections did not affect *IRS* gene expression in the brain of high and low pollen-hoarding strains or wild-type worker bees. (A) Comparison of high and low pollen-hoarding strains showed that high strain workers had higher *IRS* expression levels in brain than low strain bees. (B) Wild type. Asterisks indicate significance, p<0.05. Bars are means ± s.e. Sample sizes are given inside bars. (A) versus (B) were quantified with different calibrator samples; Y-axes cannot be directly compared.

In brain, and consistent with previous RNAi results from honey bees [47],[50], intra-abdominal injections of dsRNA did not influence the transcript level of the target gene. *IRS* expression was undisturbed in the selected strains (factorial ANOVA: treatment, F_(1, 40)_ = 0.2466, p = 0.6222), as well as in the wild type (Student t-test, T_(1, 30)_ = −0.7372, p = 0.4667). Overall, the high pollen-hoarding strain bees were characterized by higher *IRS* mRNA levels in brain than low strain workers, similar to our finding in fat body (factorial ANOVA: genotype, F _(1, 40)_ = 6.2650, p = 0.0165, Figure 2A). No treatment by genotype interaction was detected (F_ (2,40)_ = 0.2921, p = 0.5919).

#### Whole-mount *in situ* hybridization

**Figure 3:**
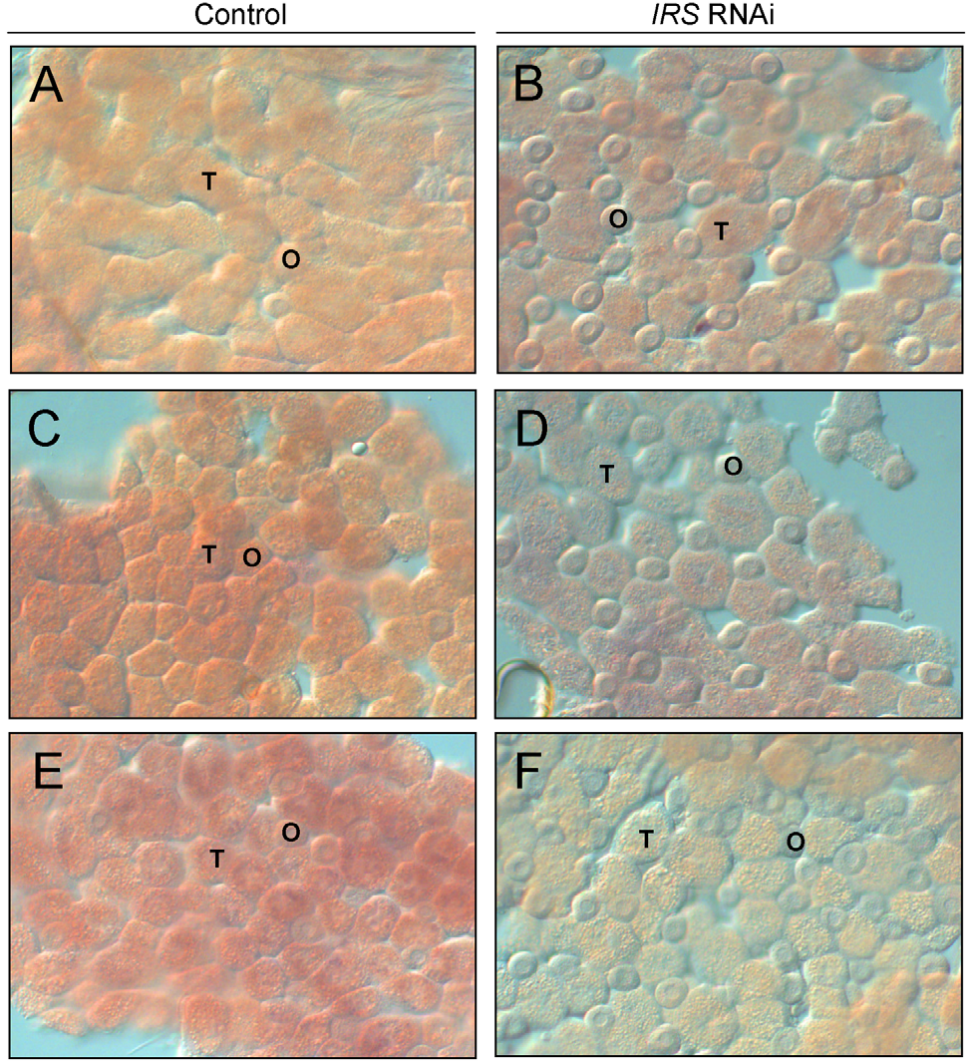
Verification of *IRS* RNAi in peripheral fat body by whole-mount *in situ* hybridization. Expression of *IRS* was confirmed in fat body, and staining intensity (purple/red color) was reduced in IRS knockdowns compared to controls. (A,B) High pollen-hoarding strain; (C,D) low pollen-hoarding strain; (E,F) wild type. The honey bee fat body is a single cell-layer primary composed of trophocytes (T) and oenocytes (O). IRS transcript was localized to both cell types. Magnification: 200×.

*IRS* RNAi in worker bee fat body was examined by *in situ* hybridization. In adult honey bees, most fat body tissue lines the abdominal wall as a single cell-layer that is primarily composed of two cell types, trophocytes and oenocytes [51]. Our analysis of abdominal fat body identified *IRS* transcript in both the trophocytes and oenocytes (Figure 3), and transcript abundance was reduced in *IRS* knockdowns compared with controls (n = 6, representative samples in Figure 3). Results were consistent in the selected pollen-hoarding strains (Figure 3A–3D), as well as in wild-type bees (Figure 3E and 3F). The effect of *IRS* RNAi in fat body, thereby, could be recognized by both qRT-PCR and *in situ* hybridization.

#### Western blot

**Figure 4:**
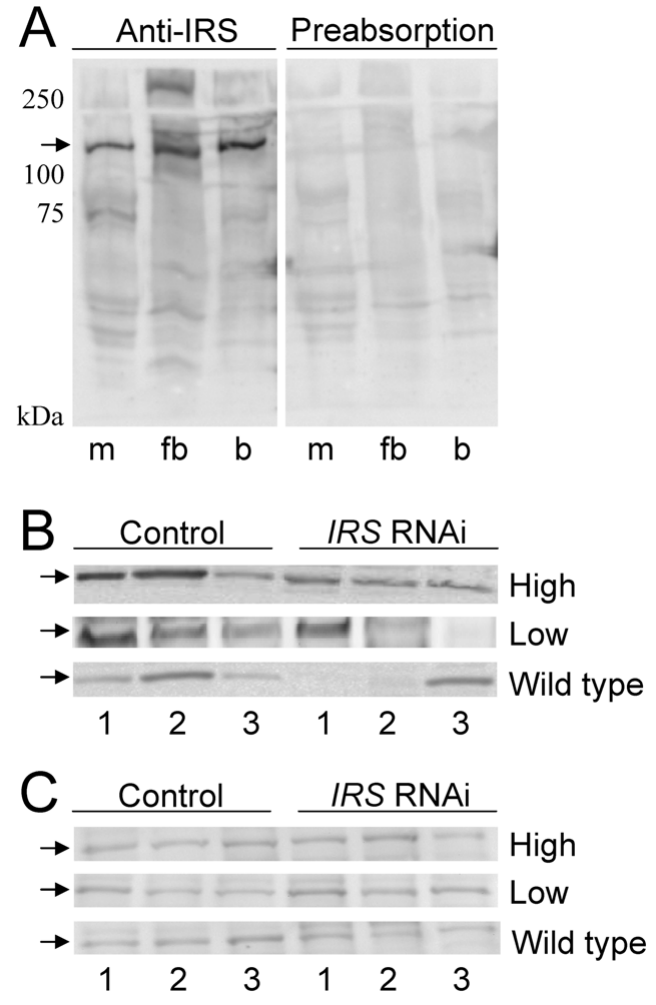
Western blot assessment of peripheral IRS knockdown. Fat body and brain protein from high and low pollen-hoarding strains and wild type was resolved on 10% SDS-PAGE gels with 100 µg protein loaded in each lane. (A) IRS antibody identifies a protein of about 130 kDa (arrow) in muscle (m), fat body (fb), and brain (b) lysates. The specificity of the antibody to the IRS peptide antigen is confirmed by preabsorption of antibody with access peptide. Detection is completely blocked by preabsorption control. (B) IRS immunoreactivity in individual protein samples from fat body (n = 3 for both treatments within each pollen-hoarding strain and wild type). The amount of the 130 kDa target protein is variable, but generally reduced after *IRS* RNAi. (C) IRS immunoreactivity in samples from brain is similar between *IRS* knockdowns and controls (n = 3 for each strain and wild type).

To examine whether RNAi-mediated *IRS* knockdown could produce bees with reduced levels of IRS protein in fat body but not in brain, we prepared a peptide antibody against honey bee IRS. Anti-IRS immunoreactivity identified one band of about 130 kDa in muscle, fat body, and brain, consistent with the predicted size of honey bee IRS (see Materials and Methods). Detection was completely blocked by preabsorption control, i.e., when antibody first was mixed with an excess amount of the IRS peptide antigen (Figure 4A). Western blot analysis of tissue samples from pollen-hoarding strains and wild-type bees suggested that the amount of IRS protein is variable in worker fat body, and that levels can be reduced by *IRS* RNAi (n = 7−8, representative samples in Figure 4B). *IRS* knockdowns and controls showed equal levels of IRS immunodetection in the samples from brain (Figure 4C), in agreement with the qRT-PCR result (above, Figure 1 and Figure 2).

### Effect of peripheral *IRS* expression on foraging choice

Using the *IRS* RNAi procedure above, *IRS* knockdown and control treatment groups were established for high and low pollen-hoarding strain bees. This experiment excluded wild-type bees, because their increased heterogeneity of genotype and behavior was anticipated to mask effects of a single gene, here *IRS*, on a complex quantitative trait like food-related behavior ([34],[48] and Discussion). All bees were marked and allowed to mature for 10 days in two host colonies. Subsequently, for five days, marked bees were captured as they returned from foraging trips and their foraging loads of pollen and nectar were quantified (n = 101 high vs. n = 168 low strain bees, further details in Materials and Methods) [32].

We identified ‘nectar load weight’ and the ‘proportion of pollen collected’ as behavioral traits that were significantly affected in the experiment. These variables were influenced by the RNAi treatment scheme (factorial ANOVA: treatment, F_(2,214)_ = 5.0528, p = 0.0071) and by strain (factorial ANOVA: genotype, F_(2,214)_ = 19.3706, p<0.0001). Host colony environment (factorial ANOVA: colony, F_(1,214)_ = 1.2328, p = 0.2935) did not affect behavior, and no interaction between treatment and genotype was detected (F_(2, 214)_ = 1.1618, p = 0.3149).

**Figure 5:**
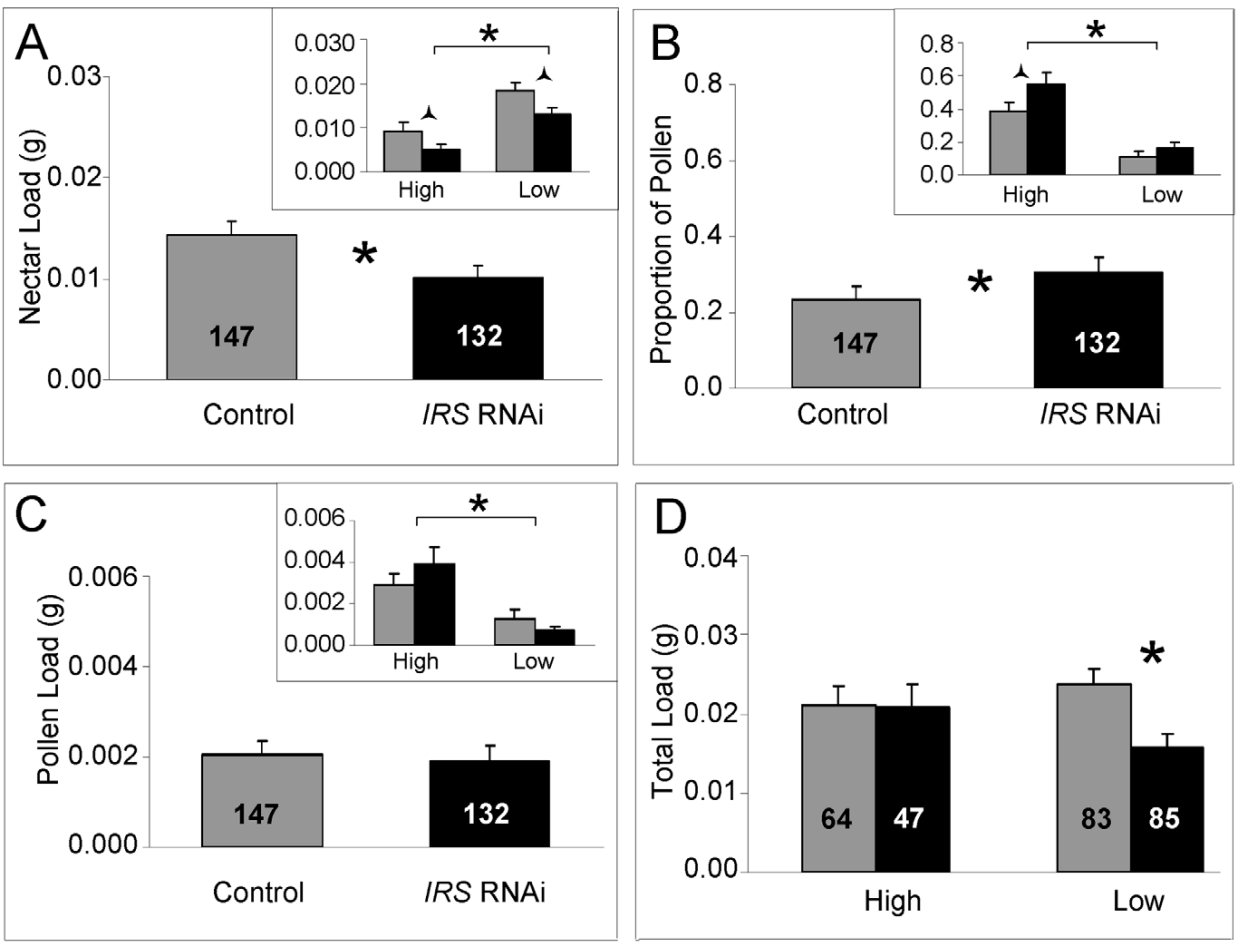
Effect of *IRS* on honey bee food-related behavior. In response to *IRS* down-regulation, worker honey bees of high and low pollen-hoarding strains (A) reduced their nectar load weight during foraging. (B) The data on proportional pollen load showed that, in comparison with controls, *IRS* knockdowns biased food-loading toward pollen. (C) Pollen load weight was not significantly affected, and only the strain effect was significant (high strain bees collect more pollen, bracket in insert). (D) The total load weight remained constant in high strain knockdowns and controls, but was reduced in low strain *IRS* knockdowns. Significant differences are indicated by asterisks p<0.05, and half (triangular) asterisks p<0.1. In all sections, brackets on top of inserts identify the strain effects on behavior. Bars are means ± s.e. Sample sizes are given inside bars.

Post hoc tests on the behavioral data were performed separately for nectar load and the proportion of pollen collected, as nectar load explains part of the variance in the proportional load of pollen [25]. The effect of *IRS* RNAi on nectar load (Fisher's LSD: p = 0.0255) and the proportion of pollen collected (Fisher's LSD: p = 0.0447) were independently significant (Figure 5A and 5B).

Further analysis showed that the behavioral response in worker nectar load weights after peripheral *IRS* RNAi remained suggestive also when the dataset was split by strain (Fisher's LSD: p = 0.0549, Figure 5A insert). These results indicated that reduced *IRS* expression in fat body affected the nectar loading behavior of the two strains similarly: nectar loads were reduced by peripheral *IRS* knockdown irrespective of genotype. Thereby, the data from both strains contributed additively to statistical power such that the significant effect of *IRS* RNAi on behavior was detected in the full dataset (Figure 5A).

For the proportional load that was pollen, a similar pattern of post hoc significance showed that both strains contributed to the significant influence of *IRS* down-regulation on behavior. This effect was observed as a consistent bias of the strains' mean foraging effort toward the pollen protein source (Figure 5B). When the data were split by strain and each set analyzed separately, the effect remained suggestive within the high strain genotype (Fisher's LSD: p = 0.0771).

### Effect of peripheral *IRS* expression on total foraging load

In our experiment, *IRS* did not affect pollen loads *per se* (factorial ANOVA, treatment, F_(1,270)_ = 0.3699, p = 0.5435, Figure 5C), but the strain effect was significant (factorial ANOVA: genotype, F_(1,270)_ = 20.94, p<0.0001; Figure 5C insert). In addition, high strain bees demonstrated a trend toward increased pollen load sizes in response to *IRS* RNAi, while the opposite was true for low strain bees (Figure 5C insert). To understand the relationships between the strain-associated pattern of pollen loading, the more general (strain-independent) effect of *IRS* on nectar load sizes (Figure 5A), and the workers' overall food-loading behavior, we analyzed the total load masses of the bees. In this analysis, the pollen load was counted twice toward the foraging effort of each worker [30],[52]. This correction of total load mass to estimate individual effort is in general use [30],[52], and takes into account that aerodynamic power influences the pollen load and nectar load of workers differently: it is possible for a forager to carry a maximum load size of nectar that is approximately twice as heavy as the maximum load size of pollen she is capable of carrying [52]. Using the raw (uncorrected) weights of nectar and pollen did not influence conclusions (Fisher's LSD test, uncorrected data, p_raw_-values in *italics*, below).

The main effects of *IRS* RNAi, strain genotype, and host environment did not affect the total foraging effort of the worker bees (factorial ANOVA: treatment, F_(1,271)_ = 2.9831, p = 0.0852; genotype, F_(1,271)_ = 2.1811, p = 0.1409; colony, F_(1,271)_ = 0.0164, p = 0.8982). However, when contrasting the loading relationships of the two genotypes in a planned comparison (Fisher's LSD test), we found that the average total load mass of high strain *IRS* knockdowns and controls was identical (Fisher's LSD: p = 0.7337, *p_raw_ = 0.5008*), while low strain bees responded to *IRS* down-regulation with a significant decrease in their total load mass average (Fisher's LSD, p = 0.0130, *p_raw_ = 0.0154*, Figure 5D). These results indicated that in response to *IRS* downregulation, increased pollen-loading (Figure 5C insert) counterbalanced reduced nectar loading in high strain bees (Figure 2A insert) while the low strain genotype collected less nectar without increasing pollen loads, leading to reduced total food-loading.

### Effect of peripheral *IRS* expression on sucrose responsiveness

The sucrose response is a general neural property related to foraging choice behavior in wild-type honey bees [53] and selected pollen-hoarding strains [20],[27],[54]. Thus, after detecting significant effects of *IRS* on foraging bias, we wanted to resolve if *IRS* knockdown influenced the workers' foraging choice by modulating the sucrose response system. To test this relationship, we quantified the effect of *IRS* RNAi on individual sucrose responsiveness measured as the gustatory response score (GRS) [20],[27],[28],[49],[53]. As before, knockdowns and controls were established and introduced into two host colonies. The experimental bees were retrieved after 11 days (n = 42−54), i.e., at a chronological age similar to the bees tested for foraging choice behavior. In the laboratory, the proboscis extension response (PER) was measured using a standard series of water and six increasing sucrose concentrations [27],[28]. Individual bees were assigned a GRS based on the number of elicited PER (0 =  lowest score, not responding to gustatory stimulation; 7 =  highest score, responding to water and all six sucrose concentrations).

**Figure 6:**
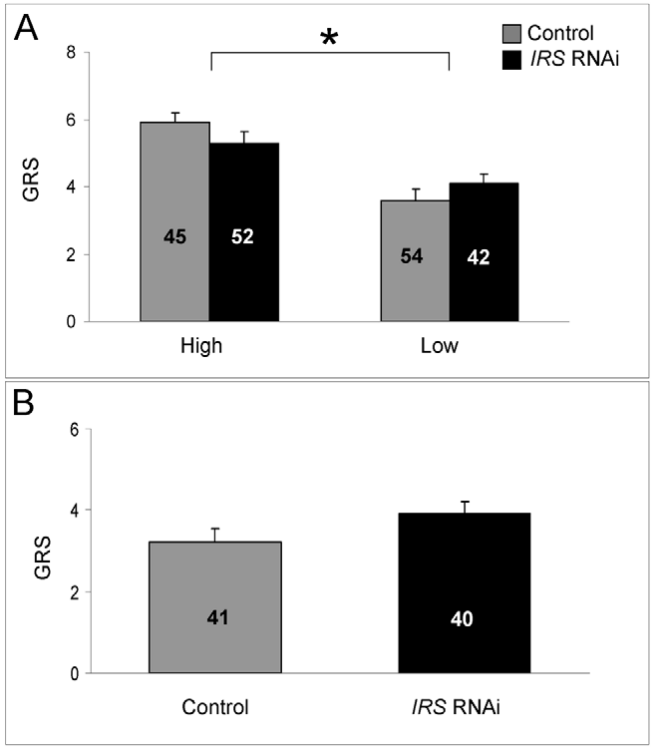
Effect of *IRS* on sucrose responsiveness. *IRS* RNAi did not influence the sucrose sensory sensitivity of (A) high and low pollen-hoarding strain bees and (B) wild type. Sucrose sensitivity was measured as a gustatory response score (GRS). High GRS implies that bees respond with proboscis extension to low concentration of sucrose in H_2_O. As established previously, high strain bees generally test higher for GRS than low strain bees [27],[28]. The strain effect is indicated by asterisk and brackets on top of panel A, p<0.05. Bars are means ± s.e. Sample sizes are given inside bars.

As shown before [20],[27],[28], we found that high strain workers were more responsive to sucrose compared with low strain bees (factorial ANOVA: genotype, F_(1,185)_ = 13.1205, p = 0.0003; colony, F_(1,185)_ = 0.88636, p = 0.3540). The sucrose response is a defining character difference between pollen-hoarding strains [16],[26],[54], and in our dataset the effect of genotype was significant in *IRS* knockdowns (Fisher's LSD: p = 0.0184) as well as controls (Fisher's LSD, p = 0.0001, Figure 6A). In contrast, *IRS* RNAi did not influence the bees' sucrose response (factorial ANOVA: treatment, F _(1,185)_ = 0.8823, p = 0.3488). There was also no interaction between the treatment and genotype factors (F _(1,185)_ = 0.7861, p = 0.3764). A validation test in wild type (n = 40−41, Figure 6B) supported that worker sucrose responsiveness is not strongly affected by reduced peripheral *IRS* expression (two-tailed Student's t-test, T_(1,79)_ = 1.6928, p = 0.0945).

### Effect of peripheral *IRS* expression on fat body *vitellogenin* mRNA level

An influence of *IRS* expression on foraging choice but not the sucrose response system of worker bees, could point to a function of *IRS* in behavioral regulation that is separate from known roles of *vitellogenin*: Honey bee *vitellogenin* encodes a multifunctional yolk protein precursor [25]. The gene is expressed in fat body and affects worker sucrose responsiveness, foraging onset, foraging choice, and lifespan [30],[49],[55]. RNAi-mediated knockdown of *vitellogenin* increases sucrose responsiveness in wild-type bees, leading to higher GRS [49]. In our experiment however, GRS remained constant despite *IRS* RNAi. This finding led us to predict that when *IRS* is knocked down, *vitellogenin* expression remains unchanged. To test this hypothesis, we measured the amount of *vitellogenin* transcript in the fat body of *IRS* knockdowns and controls (selected strains, n = 18; wild type, n = 12).

**Figure 7:**
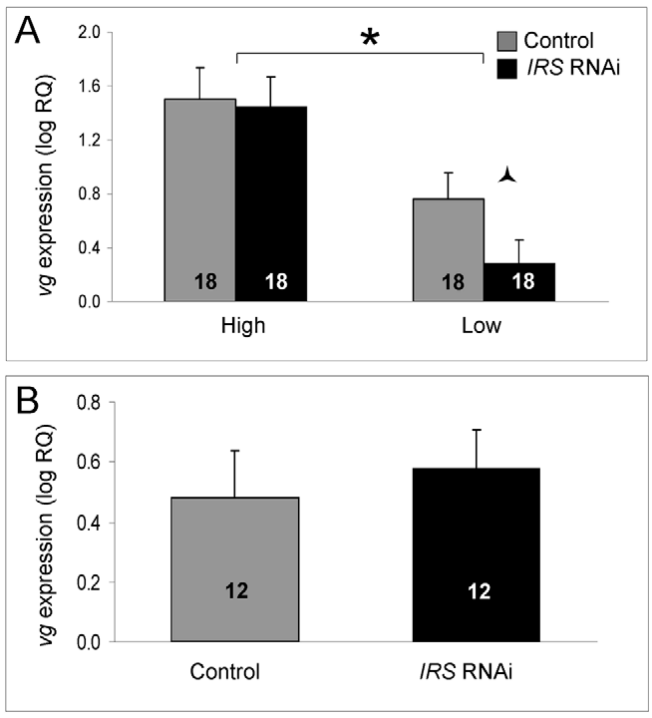
Effect of *IRS* on *vitellogenin* mRNA. *IRS* RNAi did not influence the level of *vitellogenin* gene expression overall (shown as log relative quantities (RQ), see the main text for details on statistics). However, in (A) a negative effect on the *vitellogenin* transcript level was suggestive in a planned comparison between low strain *IRS* knockdowns and controls. The *vitellogenin* expression levels of wild-type bees (B) remained unchanged. As shown before, *vitellogenin* mRNA levels were elevated in high strain workers compared with low strain bees [15],[48]. Bars are means ± s.e. Half (triangular) asterisk indicates p<0.1. Brackets and asterisk on top of (A) denote the strain effect, p<0.05. Sample sizes inside bars. (A) versus (B) were quantified with different calibrator samples; thus, Y-axes cannot be directly compared.

As established previously, the level of *vitellogenin* mRNA was significantly different between high and low pollen-hoarding strain bees (factorial ANOVA: strain, F_(1,59)_ = 14.3995, p = 0.0004). Young (less than 15 day-old) high strain workers are characterized by elevated *vitellogenin* expression levels compared to same-aged low strain bees [15],[48]. In our experiment, this pattern was confirmed in the data from *IRS* knockdowns (Fisher's LSD, p = 0.0202) and controls (Fisher's LSD, p = 0.0003, Figure 6A). Moreover, and as predicted, *IRS* RNAi did not influence the amount of *vitellogenin* transcript overall (factorial ANOVA: treatment, F_(1,59)_ = 0.9660, p = 0.3297). A planned comparison in each strain (Student's t-test), however, indicated that low pollen-hoarding strain bees tend to reduce *vitellogenin* expression after *IRS* RNAi (T_(1,31)_ = 1.8274, p = 0.0386, Figure 7A). This response was not paralleled in high strain workers (T_(1,32)_ = 0.1637, p = 0.4355). Wild-type (Figure 7B) also did not show an effect of *IRS* RNAi on *vitellogenin* (two-tailed Student's t-test, T_(1,22)_ = −0.1720, p = 0.8650).

## Discussion

Here, we show that *IRS* can affect the foraging decisions of an insect. Knockdown of peripheral *IRS* gene expression led 10–15 day-old worker honey bees of two standard genetic backgrounds to collect less nectar and to bias their foraging effort toward pollen. The effect of *IRS* on foraging behavior was subtle but significant. Modest influences are the common denominator of intrinsic behavioral affectors in worker bees, including the *vitellogenin* gene [30], the TOR (target of rapamycin) signaling pathway, and fat body adiposity [35, and references therein]. Worker behavioral traits, including food-related task performance, are complex quantitative genetic characters [31]–[34] that also are modulated by social environmental factors like the amount of larval brood and stored food-resources in colonies [19]. Many genes that influence honey bee behavior, therefore, may not have major effects [24].

Before obtaining behavioral data, we validated RNAi in 7 day-old bees (Figure 1, Figure 2, Figure 3, Figure 4). *IRS* mRNA and protein levels were measured in these workers and not in the bees from our behavioral experiment, because transcript abundance can be influenced (and thus confounded) by the considerable laboratory handling that is required for collection and quantification of honey bee foraging loads. Yet, RNAi can last up to 25 days in honey bee workers [30],[46], and more than 4 months in the flour beetle *Tribolium castaneum* [56]. In other insect species, such as aphids [57],[58] and termites [59], RNAi-mediated gene-silencing is not as long-lasting, but the transient effect is sufficient to induce enduring changes in life-history. Thus, the cumulative evidence from insect functional genomics, in combination with our treatment-specific results, strongly suggests that *IRS* RNAi persisted beyond the 7^th^ day validation point.

We used wild-type honey bees to validate the RNAi tool, and to show that connections between *IRS* and sucrose response, and between *IRS* and *vitellogenin* expression, could be generalized. Yet, only the standard stocks of high and low pollen-hoarding strains were used to test whether knockdown of peripheral *IRS* expression could influence foraging behavior. Honey bee foraging choice is a complex quantitative trait: it is governed by many genes and some loci are highly epistatic [34]. Effects on behavior might be undetectable if one gene in such networks is perturbed within a highly heterogeneous group of animals, like wild-type honey bees. Wild-type colonies differ in levels of pollen-hoarding, and wild-type workers show variation in food-related behavior [17],[25]. Within each of the standard pollen-hoarding strains, such variance is present but reduced, and the well-documented differences between the genotypes can be controlled for so treatment effects are more easily detected [48]. Therefore, we assumed that the effects of peripheral *IRS* RNAi were more likely to be revealed by using these two standard genetic backgrounds in our test of behavior.

Artificial selection can result in spurious phenotypic associations, and it can be relevant to ask whether results from selected stocks can be generalized to unselected animals (wild type) [60]. Pollen-hoarding has affected a suite of traits in worker bees, including sucrose responsiveness (Figure 6), and *vitellogenin* expression (Figure 7), in addition to behavior [15],[17],[24],[27],[48]. The majority of these trait-associations are tested and verified to extend to wild type [16],[24],[25],[29]. Thus, it is likely that a set of worker traits including food-related behavior are pleiotropically regulated, and that the underlying gene network responded to artificial selection on pollen-hoarding [61]. This network can be represented in the *pln1*-*pln4* QTL, where *IRS* is a positional candidate gene. The *pln* network has been mapped in different genetic sources of honey bees, which suggests that it is generally important for worker behavior [34]. Genetic background, however, affects both gene expression (as shown in Figure 1, Figure 2, and Figure 7) and behavior (as exemplified in Figure 5 and Figure 6), and thereby, our data on foraging behavior are not generalizable. Yet, the contributions from this study do not only draw from an ability to generalize to wild-type bees. Rather, the results serve as a first illustration of a role of peripheral *IRS* in behavioral control.

We identify a behavioral outcome of *IRS* down-regulation that is independent of genotype: the increased preference for a protein source (pollen). However, we also reveal that the behavioral bias toward protein can be achieved through genotype-specific behavior. The strain selected for a high level of pollen-hoarding responded to reduced peripheral *IRS* expression by collecting smaller nectar loads and larger pollen loads than controls, resulting in a significant increase in the proportion of pollen collected. Overall, the total food load did not change. The strain selected for a low level of pollen-hoarding, on the other hand, did not compensate for reduced nectar loading by collecting more pollen. Thereby, the total food load declined.

We propose that these behavioral responses can be explained if the foraging choice behavior of the worker honey bees is jointly influenced by fat body *IRS* and *vitellogenin* expression. Our explanation builds on three insights; that the total load mass of bees has an upper limit during foraging and therefore nectar vs. pollen loading is negatively correlated [52]; that *vitellogenin* expression encourages pollen loading [15],[25],[30], and, that vitellogenin protein may reduce IIS transduction [55],[61],[62]. Explicitly, workers decrease nectar loading in response to *IRS* down-regulation (Figure 5A), and in the presence of high *vitellogenin* levels available loading-capacity fills up with pollen (high strain, Figure 5, Figure 7A). The general pollen bias of high strain bees is consistent with this explanation, as the higher intrinsic vitellogenin level of this genotype would reduce IIS transduction and encourage pollen loading also in unmanipulated workers. In the low strain, conversely, lower intrinsic vitellogenin levels may normally encourage IIS transduction and nectar loading. And, when *IRS* is artificially suppressed in conjunction with low (and further declining) levels of *vitellogenin* expression (our experiment), reduced nectar loading is not counterbalanced by release of pollen foraging behavior. As a result, the total load mass declines (low strain, Figure 5, Figure 7A).

Vitellogenin is a glyco-lipoprotein that may convey a general signal of fat body adiposity [63]. In *Drosophila*, central IIS can be regulated remotely by nutrient sensing in fat body cells, but increased nutrient availability is associated with increased IIS in the fly [44]. The inverse influence of nutrition (or vitellogenin action) on IIS in honey bees is under study but poorly understood [55],[61],[62]. Correlations in our data may add to this investigation: high strain bees have high *vitellogenin* transcript abundance and somewhat increased *IRS* mRNA levels compared with low strain bees (Figure 1A, Figure 7A). These relationships could imply that vitellogenin does not influence IIS by reducing *IRS* expression. It remains to be tested whether the elevated amount of *IRS* transcript in high strain bees is a compensatory response to reduced IIS transduction.

Manipulation of IIS pathways can disrupt energy homeostasis and metabolism and produce extreme hyper- and hypoglycemic states leading to changes in food-related behavior [39],[64]. Could similar processes influence our results? In *Drosophila*, circulating blood sugar levels increase if ILP secretion is suppressed [65]. However, mutations in the fly *IRS* gene homologue *chico* lead to elevated lipid levels but the amount of circulating carbohydrate is unchanged [66]. Indeed, it has been suggested that ILPs may not be primary regulators of glucose homeostasis in insects: Adipokinetic hormone (AKH), an endocrine factor with functions similar to glucagons, may govern global carbohydrate levels instead [67]. Thus, if energy homeostasis is similarly controlled in honey bees and fruit fly, it is less probable that the behavioral changes we observe here result from non-physiological hyperglycemia. This conclusion is supported by general results from high and low pollen-hoarding strain bees, which do not differ in baseline blood glucose levels or in blood glucose response to diets of varying sugar concentration (Supplementary Figure 1 in Text S1). The genetic differences between the strains (which likely influence IIS processes [34]), thereby, may not confer measurable differences in glucose homeostasis.

Many questions remain unanswered about how nutrients, vitellogenin, and IIS modulate physiology and behavior in honey bees. In this context, the work presented here represents the first successful gene knockdown of a central and conserved IIS pathway gene, and provides the first look at consequences for behavior. The honey bee is a study system in metabolic biology, sociobiology, behavioral biology, and neuroscience [50]. Thus, in addition to revealing a role of *IRS* in worker foraging behavior, our results provide tools for research on how life-histories are affected by metabolism, brain chemistry, and social behavior.

Like eating behavior in mammals, foraging behavior in the honey bees is a complex syndrome influenced by genotype, physiological state, environment, and social needs. Much remains to be discovered about the behavioral physiology of food choice. This research is a priority as obesity-related disorders claim an increasing human health and economic toll. Our data are first to show that peripheral *IRS* expression can influence an insect's foraging choice between protein and carbohydrate sources. This finding sets the stage for comparative work that can increase our knowledge on the biology of food-related behavior.

## Materials and Methods

### Bees

Bees were maintained at the Honey Bee Research Laboratory at the Arizona State University Polytechnic Campus. Two high pollen-hoarding strain colonies, two low pollen-hoarding strain colonies, and two wild-type colonies were used as donors of experimental workers. To obtain the bees, queens were caged on a wax comb and allowed to lay eggs for 24 h inside the colony. Subsequently, the combs were removed and marked according to source before the brood was co-fostered in wild-type colonies. After 20 days, the combs were collected and put in an incubator where the bees emerged at 34°C and 80% relative humidity.

### Cloning of *IRS*

The most recent honey bee genome assembly identifies XM_391985 as *IRS*. In a former genome release (version 3), *IRS* was identified as GB11037-RA, which differed from XM_391985 by the presence of an extra exon. We cloned *IRS* from total RNA isolated from several adult tissues (worker brains, fat bodies, and ovaries) to capture putative alternate splicing of the gene. Forward and reverse primer 5′ CACAACCGCAATCTCAGTC 3′; 5′ AACATAGTCGGCAGGTGGAC 3′, respectively, were used. Four independent clones from each tissue were sequenced. The data confirmed that XM_391985 is a correct sequence for *IRS*, and did not detect alternative splicing.

To produce cDNA template for double stranded RNA (dsRNA) synthesis, a 700 bp fragment from the open reading frame of the *IRS* (XM_391985) mRNA sequence was cloned by forward and reverse primer 5′-TTTGCAGTCGTTGCTGGTA-3′; 5′-GCTTAAAGCCGGATAACGTG-3′, respectively, into pCR® 4-TOPO® vector using the TOPO TA cloning kit (Invitrogen). Cloning followed the instructions provided by the manufacturer. Several clones were verified by sequencing.

### Preparation of dsRNA

For dsRNA synthesis, PCR primers with T7 promoter sequences (underlined) were used. The cloned cDNA fragment was used as a template for PCR, with 5′-TAATACGACTCACTATAGGGCGAGCGAACCGGTAGTCGTAAAG-3′ and 5′-TAATACGACTCACTATAGGGCGAGCAGTGATCAAACGTGGCTT-3′ as forward and reverse primer, respectively. The resulting product was 583 bp long. As control, green fluorescent protein (GFP) dsRNA was synthesized from AF097553 template, as previously described [46],[48],[49]. PCR products were excised from low melting temperature 1% agarose gels, purified using Qiaquick Gel Extraction Kit (Qiagen). The dsRNA was then made using AmpliScribe T7 transcription kit (Epicentre Biotechnologies) following the manufacturer's protocol. dsRNA was purified using phenol:choloform extraction and run on a 1% agarose gel for verification of size and purity [58]. The final dsRNA concentration was adjusted to 10 µg/µl in nuclease free H_2_O.

### Preparation of samples for *IRS* knockdown validation

Newly emerged workers (high and low pollen-hoarding strains plus wild type) were randomly assigned treatments and marked with paint (Testors Enamel, Testor Corporation) to indicate treatment identity. Treated bees were injected intra-abdominally with either dsRNA against the *IRS* gene or, with green fluorescent protein (GFP)-derived dsRNA to establish a control, following general procedures for knockdown of gene expression in honey bee fat body [30],[46],[49]. The injection volume was 3 µl. After dsRNA injection, bees were introduced into two host colonies with a background population of about 5,000 wild-type bees. Fat bodies and brains were dissected from 7 day-old marked bees, and tissues flash-frozen in liquid nitrogen and stored at −80°C until use.

### qRT–PCR

RNA was extracted using RNeasy Mini Kit (Qiagen) including DNase treatment. For mRNA quantification between control and *IRS* knockdown workers; two step (real time) qRT-PCR was performed in triplicate using ABI Prism 7500 (Applied Biosystems), and the data were analyzed using the Delta-Delta CT [68] method with *actin* (GenBank: XM_623378) as housekeeper gene. This gene is stably expressed in different honey bee tissues, and provides a reference for studies of gene expression in the bee [69],[70]. By monitoring negative control samples (without reverse transcriptase) and melting curves, we could verify that the qRT-PCR assay was not confounded by DNA contamination or primer dimmers [71].

### Whole-mount *in situ* hybridization

*In situ* hybridization was performed according to a modified protocol based on Osborne and Dearden [72] and optimized for honey bee fat body. Fat bodies were fixed in buffer (4% formaldehyde, 20 mM KH_2_PO_4_/K_2_HPO_4_, pH 6.8, 90 mM KCl, 30 mM NaCl, 4 mM MgCl_2_) [73] at 4°C overnight with shaking, then washed three times in PBS. The samples were dehydrated through a methanol series and stored in methanol at −20°C. Rehydration was accomplished with a methanol series and followed by PTw washes (PBS +0.1% Tween-20).

Fat bodies were digested with 20 µg/mL Proteinase K for 15 min, rinsed in PTw, and postfixed for 15 min in PTw with 4% formaldehyde. After rinsing five times in PTw, samples were transferred to 500 µl of hybridization buffer (50% deionized formamide, 5×SSC, 1 mg/ml yeast tRNA, 100 µg/ml salmon sperm DNA, 100 µg/ml heparin, 1xDenhardt's Solution, 0.1% Tween 20, 5 mM EDTA) and prehybridised at 60°C for 2 h.

Hybridization was conducted in a hybridization buffer with 2 ng/µl specific *IRS* RNA probe labeled with digoxigenin (DIG). To remove unbound probe, fat bodies were washed at 60°C in each of a series of pre-warmed wash solutions for 30 min in the order [74]: 75% hybridization buffer +25% 2×SSC, 50% hybridization buffer +50% 2×SSC, 25% hybridization buffer +75% 2×SSC, 100% 2×SSC, 0.2×SSC. Then, the samples were washed at room temperature 10 min in the following solutions: 75% 0.2×SSC +25% PTw, 50% 0.2× SSC +50% PTw, 25% 0.2× SSC +75% PTw, 100% PTw.

The samples were blocked with 0.1% sheep serum in PTw for 20 min at room temperature, followed by incubation with a 1∶ 2,000 dilution of Anti-DIG-alkaline phosphatase conjugated Fab fragments (Roche Molecular Biochemicals) in blocking buffer at 4°C overnight. Tissues were then washed three times in alkaline phosphatase buffer (1 h). The color reactions were developed by BM purple alkaline phosphatase substrate precipitating at 4°C overnight. Reactions were stopped by dilution in PTw.

The color reactions were developed by BM purple AP substrate precipitating at 4°C overnight. Reactions were stopped by dilution in PTw. The samples were visualized on an upright microscope (Axio Imager A1, Carl Zeiss Microimaging) at 200× magnification and photographed (Axiocam MRc5, Zeiss Microimaging).

### Protein extraction and western blot

Fat body and brain tissues were ground for 1 min in 150 µl and 50 µl extraction buffer, respectively (20 mM Tris, 150 mM NaCl and 5 mM EDTA) supplemented with protease inhibitor (Complete, Mini Protease Inhibitor Cocktail Tablets; Roche Applied Science) on ice. Samples were centrifuged at 6,000×g for 20 min, and the supernatant was transferred into a new tube. The total protein concentration in this fraction was quantified using Bradford reagent [75]. Aliquots of individual samples, each with 100 µg protein, were then subject to SDS-PAGE on 10% gels (Promega) and transferred onto PVDF membrane (Bio-Rad). Non-specific protein binding was blocked with 3% instant non-fat dry milk (BestChoice) overnight.

Preabsorption was used to determine the specificity of the antibody toward IRS peptide antigen. Briefly, purified antibody (1.5 µg/ml) and the antigen peptide (0.2 µg/ml) were mixed in the blocking solution (3% Milk solution in 1XPBST) in a total volume of 15 ml, and incubated on a rocking platform for 1 h. Membranes were probed either with this preabsorption solution for 1 h, or with purified IRS antibody (1∶500) in 15 ml blocking solution for 1 h. These incubations were followed by 3 washes with 1XPBST at 10 min interval. Membrane-bound antigen-antibody complexes were visualized with horseradish peroxidase-conjugated goat anti-rabbit IgG (GE healthcare) at a dilution of 1∶1,000 and detected with Western Lightning Chemiluminescence reagent (PerkinElmer) on a Versa-Doc imaging system (Bio-Rad). IRS immunoreactivity identified a band of about 130 kDa, similar to the predicted molecular weight of honey bee IRS (129 kDa, Protein Calculator v3.3, http://www.scripps.edu/~cdputnam/protcalc.html).

### Foraging preference

dsRNA injections took place over two days for both of two experimental colonies, following the procedures described above. For every colony replicate, we prepared 150 bees from each treatment group and pollen-hoarding strain. All bees were marked with paint to indicate treatment group identity (*IRS* RNAi or GFP control) before they were introduced into the nests. Each experimental colony had a background population of about 5,000 wild-type bees. The experimental bees were allowed to mature. When bees from both treatment groups and genotypes were observed returning from foraging trips (after 10 days), collection of foragers was initiated. Foragers were collected over a five-day period during peak foraging hours [30]. Pollen loads were removed from the left corbicula and weighed. We expelled the nectar from foragers' honey stomachs into pre-weighted capillary tubes to measure nectar load weight with a digital balance as described before [21],[53]. Sucrose concentration was measured using a digital refractometer (Misco).

### Measuring gustatory responsiveness and *vitellogenin* mRNA expression

The same protocols for dsRNA injection (n = 100) and sample collection (above) were used to obtain treatment and control workers for the measure of gustatory responsiveness and *vitellogenin* transcript levels. The 11 day-old high and low strain bees were collected in the morning and placed individually in the cylindrical mesh cages. Each bee was chilled until it showed first signs of immobility. It was then mounted in a metal holder and fixed with two strips of adhesive tape between head and thorax and over the abdomen [76].

After 1 h, gustatory responsiveness was tested using the proboscis extension response (PER). The investigator was blind to the treatment identity of the bees. Each worker was tested by touching both antennae with a droplet of H_2_O followed by a concentration series of 0.1, 0.3, 1, 3, 10, 30% sucrose. The inter-stimulus interval was 5–7 min. The interval was variable with the number of individuals tested at one time, usually 40–60 bees per test. A bee was observed to ‘respond’ to stimulation by fully extending its proboscis when a drop of water or sucrose was touched in turn to each antenna. The sum of the responses elicited during the test series represented the gustatory response score (GRS) of the bee [54].

After ending the test, the bees were assessed for their response to honey. Bees that did not respond to honey were not used in the subsequent data analysis, because we could not exclude that these workers were in poor condition or dead. For the remaining bees, GRS ranged between 0 (response to honey, but no response to H_2_O and any of the sucrose solutions) and 7 (response to all solutions including H_2_O).

For quantification of *vitellogenin* gene expression, mRNA was extracted from a parallel set of worker bees. As for *IRS*, qRT-PCR was used to quantify *vitellogenin* transcript levels in fat body tissue (details above on the qRT-PCR procedure). Forward and reverse primer was 5′-GTTGGAGAGCAACATGCAGA-3′; 5′-TCGATCCATTCCTTGATGGT-3′, respectively.

### Statistics

The *IRS* gene expression data were log-transformed to approximate normality [70],[77]. The resulting values conformed to assumptions of ANOVA as assessed by normal probability plots of residuals was well as by Bartlett and Levene's tests for the homogeneity of variances. A factorial ANOVA was used to validate the efficacy of RNAi. The behavioral data on nectar loads were square root transformed. A factorial ANOVA was used for initial exploration of the data on foraging behavior, which passed examination of normal probability plots on the residuals of the analysis, and also the homogeneity of variances tests (Bartlett, Levene). Yet, the variables for foraging load are not independent: when a worker collects more nectar her pollen loading-capacity is reduced, causing nectar and pollen load-weights to be negatively correlated. Thus, separate main effects ANOVA's were used for the subsequent tests. Post hoc analyses were performed with the Fisher LSD test. Factorial ANOVA and Student's t-test were used for the study of GRS scores and *vitellogenin* gene expression (log-transformed transcript levels), as the datasets conformed to assumptions of parametric tests (see above). One-tailed tests were used when appropriate, i.e., if an *a prior* expectation was established. All analyses were performed with STATISTICA 6.0 (StatSoft).

## Supporting Information

Text S1 Hemolymph glucose measurements: materials, methods, and results for quantification of hemolymph (blood) levels of glucose in high and low pollen-hoarding strain bees.
(0.17 MB PDF)
