# Supplementary material for: Down-Regulation of Honey Bee IRS Gene Biases Behavior toward Food Rich in Protein
Source: PLoS Genet. 2010 Apr 1;6(4):e1000896. doi: 10.1371/journal.pgen.1000896 (PMC2848551; doi:10.1371/journal.pgen.1000896)
Supplement: Text S1 — Hemolymph glucose measurements: materials, methods, and results for quantification of hemolymph (blood) levels of glucose in high and low pollen-hoarding strain bees. (0.17 MB PDF) [file pgen.1000896.s001.pdf]

Supporting Information
Hemolymph Glucose Measurements

Materials and Methods

Bees. Newly emerged worker bees were obtained from wax combs kept in an incubator at 34 ºC and 80% relative humidity. Combs were collected from 9-10 separate colonies from each of the high and low pollen-hoarding genotypes.

Baseline glucose titer at emergence. From every colony source, three bees were picked as they emerged from the comb (i.e. before they could feed). Bees were bled for 1.0 µl hemolymph (blood) through an incision in the abdominal wall. The combined 3.0 µl of hemolymph were pooled to form one biological sample, representing that colony. This approach gave 9-10 biological samples per genotype. The same experimental setup was run as an independent replicate. However, instead of collecting one biological sample per colony, three biological samples were obtained. This resulted in a total number of hemolymph pools (over the two replicate experiments) of 36-40 per strain. For quantification, 35 samples were analyzed for each genotype.

Glucose titer of bees fed different sucrose solutions. From every colony source, five newly emerged bees were placed into each of two laboratory cages after first receiving a genotype-specific paint mark on the thorax. One cage received 30% sucrose in H_2O; the other received 50% sucrose in H_2O. In total, four replicate cage-pairs were prepared and incubated at 34 ºC with 80% relative humidity. Sucrose solutions were replenished daily and water was given in a separate feeder. After four days, bees were obtained for hemolymph collection. Again, 1.0 µl hemolymph was pooled from each of three bees to form a biological sample. Ten biological samples were collected from each genotype and cage, resulting in a total sample size of 40.

Glucose quantification. Glucose Assay Kit (Sigma Aldrich) was used to measure the glucose concentration in hemolymph following the manufacturers' instructions. This

procedure was used previously to measure glucose levels in *Drosophila melanogaster* [1]. Each sample was run in duplicate.

Statistics. The data conformed to assumptions of ANOVA, as established by normal probability plots on residuals as well as Levene and Bartlett's tests on the variances. For newly emerged bees, we used main effects ANOVA to test the effect of strain (independent variable) on glucose levels (dependent variable), while controlling for cage and replicate effects. Cage and replicate did not influence the results ($p>0.1$). This ANOVA model was also used on the data from bees fed 30% vs. 50% sucrose solutions, with strain and sucrose treatment as independent variables. Because cage and replicate did not influence the analysis ($p>0.1$), the data were tested further by factorial ANOVA to account for interactions between strain and sucrose treatment.

Results

At emergence, the hemolymph titer of the worker bees was similar (main effects ANOVA: $F_{(1,139)} = 0.0180$, $p = 0.8934$, Supporting Figure S1A). Strain and sucrose concentration also did not influence the hemolymph glucose titer after four days (main effects ANOVA: strain, $F_{(1,152)} = 0.5346$, $p = 0.4658$; treatment, $F_{(1, 152)} = 2.5679$, $p = 0.1111$). This result was confirmed by factorial analysis (factorial ANOVA: strain, $F_{(1,151)} = 0.4920$, $p = 0.4841$; treatment, $F_{(1,151)} = 2.4227$, $p = 0.1217$; strain x treatment, $F_{(1,151)} = 2.6384$, $p = 0.1064$, Supporting Figure S1B). A planned comparison of high strain bees fed 30% vs. 50% sucrose in H_2O, however, suggested that the sucrose treatment schedule might have weakly influenced glucose levels in this genotype (Fisher's test: $p = 0.0236$).

References

1. Flatt T, Min KJ, D'Alterio C, Villa-Cuesta E, Cumbers J, Lehmann R, Jones DL, Tatar M, 2008. *Drosophila* germ-line modulation of insulin signaling and lifespan. Proc Natl Acad Sci USA 105: 6368-6373.

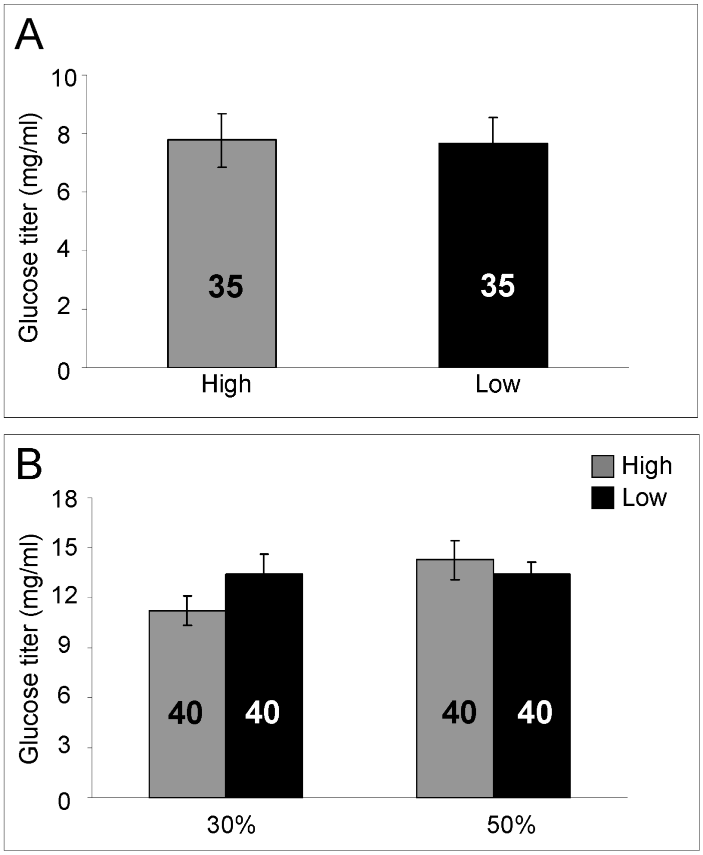

Supporting Figure S1. Circulating hemolymph glucose titer in high and low pollen-hoarding strain bees. (A) The baseline titer of newly emerged worker bees; (B) titer levels after four days of feeding with 30% or 50% sucrose in H_2O. Bars are means ± s.e. Each biological sample consists of pooled hemolymph from three individual bees. Sample sizes are given inside bars.
